# Diurnal variations in milk macro-mineral concentrations in Holstein dairy cows in Urmia, Iran

**Published:** 2012

**Authors:** Shahram Nozad, Ali-Gholi Ramin, Siamak Asri Rezaie

**Affiliations:** *Department of Clinical Sciences, Faculty of Veterinary Medicine, Urmia University, Urmia, Iran.*

**Keywords:** Milk, Cows, Milking time, Holstein, Macro-minerals

## Abstract

Milk samples from high and low milk producer Holstein cows, were obtained during the morning and afternoon milking over a one week period. Overall, 1064 samples were tested within 14 times sampling in Urmia, Iran. Milk macro-mineral concentrations in the morning milking and in low producers were greater than in the afternoon and in high producers. The highest and lowest concentrations were observed in Na^+^ and Mg^++^, respectively. Mean milk values between low and high producers in the morning, afternoon and daily milking times were different (*p *< 0.05). The individual comparison of milk parameters between both groups in the different milking times were also different (*p* < 0.05). The results of correlation among macro-minerals in the morning, afternoon and overall milking showed significant and positive correlations among all macro-minerals except for Na^+^ and K^+^, in which there was a significant negative correlation (*p *< 0.05). The highest and lowest correlations were found between Ca^++^ and inorganic phosphorus (IP) (r=0.37, *p *< 0.05) and Na^+^ and IP (r=0.10, *p *< 0.05), respectively. It is concluded that the concentration of macro-minerals in different producers varied between milking times. The sodium concentration was the highest while Mg^++^ was the lowest among macro-minerals. The correlation between Ca^++^/ IP was the highest, while Na^+^/K^+^ revealed a negative correlation. Therefore, by organizing the appropriate macro-minerals in the ration, it would be possible to achieve an optimal purpose from animal husbandry.

## Introduction

Among ruminants, cow's milk is known to be the healthiest, most widely available and cheap, and is widely consumed in both animal and human children, due mainly to the vast minerals in its composition. Milk minerals are considered the main inorganic composition, as they participate in bone growth, stability and formation of bone matrix in all consumers, specifically young newborn animals. Cow's milk contains 8 to 9 g L^-1^minerals including macro and trace minerals and has been investigated by many authors.^1^ Over 80% of milk macro-minerals are soluble in milk serum, which is possible to assess, and remaining 20% is conjugated by milk casein and is not considered as part of the macro-mineral values in the laboratory evaluation. The concentration of Mg^++^ in milk is lower than the other macro-minerals. The main roles of macro-minerals in milk are considered, as they provide the Ca^++^ and Mg^++^ for bone growth and weight gain in young animals, casein denaturizing in abomasum, and prevention of metabolic and nutritional disorders among both young and old animals.^2^^,^^3^

The mean concentrations of Ca^++^, inorganic phosphorus (IP), Mg^++^, Na^+^ and K^+^ in milk serum were reported as 48.40, 30.40, 5.42, 10.80 and 58.50 mmol L^-1^, respectively.^1^ These values are influenced by lactation period, breed and milk yield,^4^^,^^5^ milk urea and protein, heat stress and season and estrus period. 

Nowadays, the interrelationships between macro-minerals in blood serum,^6^ the musculoskeletal system, cerebrospinal fluid (CSF)^3^ and urine are confirmed and published elsewhere, although not for milk serum. Gaucheron reported the probable partial correlations among milk macro-minerals.^7^ Some authors believe that Ca^++^ absorption from milk in gut and the occurrence of calf hypomagnesaemia is directly dependent on the amounts of milk Mg^++^,^2^ and finally, correlations between milk Ca^++^, K^+^ and Na^+^ are found only in high producer cows.^8^

This information reveals that many factors such as the amount of milk yield, milking times, breed, season, and diet influence the macro-mineral concentrations in consumed milk. Meanwhile, the high level of minerals in milk is considered an appropriate quality of milk and this mineral balance results in an increase in gut absorption of minerals, which will lead to the prevention of rickets, calf tetany and retarded growth rate. To understand the effects of milk volume and milking times on macro-minerals we determined to study variations in milk macro-minerals in the morning, afternoon and daily milking in Holstein dairy cows, to compare the diurnal variations of milk parameters in moderate and high producer cows and to present the relationships among milk macro-minerals in dairy cows.

## Materials and Methods

Seventy six dairy Holstein milking cows, including 36 cows with a moderate milk yield up to 20 kg per day and 40 cows with high milk yield up to 40 kg per day were selected in 2010 in the northwest of Iran. The average milk yield in the selected industrial dairy herd was from 29 to 32 kg per day. The high producer cows were all in the first half of lactation period and the majority of moderate producers were in the second half of lactation. Ten mL milk samples in the morning and afternoon milking were collected for seven days. Overall, 1064 milk samples were collected within 14 times and the samples tested for undergoing milk parameters. During sampling, the cow's ear identification number was recorded to determine the diurnal macro-mineral variations in each cow within a one-week period. Age, pregnancy, number of parity and daily milk yield were recorded for further studies. Cows were fed *ad libitum* diet containing lucerne, pulp, concentrate and silage, four times per day. During the study mastitis and other clinical diseases were assessed and the cows were all found to be in good health. 

Milk samples were first defatted by placing them in a cool area (4 ˚C) and/or centrifugation in 3000 *g* for 5 minutes. Milk casein was separated by 0.1 N HCl in pH 3.6. Milk serums were used to determine the macro-mineral concentrations. Calcium, IP and Mg^++^ concentrations were measured using appropriate kits (Pars-Azmoon Co., Tehran, Iran) in an auto-analyzer (RA-1000, Pharmacia Co., LKB, Novaspec, USA). Milk Na^+^ and K^+^ concentrations were assessed by a flame photometer (Jenway PFP-7, Essex, UK) using standard Na^+^ and K^+^ (Ziest Chimi Diagnostics, Tehran, Iran). 

Data were analyzed by SPSS statistical software package (version 13, SPSS Inc., Chicago, IL, USA) and mean ± SEM were determined for milk parameters in the morning, afternoon, daily and overall milking yield. Student *t*-test and one way ANOVA were carried out to find out the differences in the parameters under study for each milking yield. Pearson correlation tests were used to evaluate the relationship among parameters in different milking yields. A *p*-value at least less than 0.05 was considered to be significant.

## Results

Mean macro-mineral concentrations in both producer cows (except for Na^+^ in high producer cows) were greater in the morning than in the afternoon milking (Table 1). Mean milk macro-mineral concentrations in the moderate producers were greater than in the high producer cows. Mean daily macro-mineral concentrations were 13.25, 10.76, 3.84, 76.20 and 17.10, respectively, in which the Na^+^ concentration was greater and the Mg^++^ was lower than the other concentrations (Table 1).

**Table 1:** Comparison of mean ± SEM of the milk micro-mineral concentrations (mmol L^-1^) in the morning (n=495), afternoon (n=512) and daily milk (n=1007) in the moderate and high producer cows

**Parameters**	**Calcium**	**Phosphorus**	**Magnesium**	**Sodium**	**Potassium**
**Moderate producers (Morning milking)**	13.49 ± 0.15^a^	11.21 ± 0.22^a^	3.99 ± 0.06^a^	77.10 ± 2.34^a^	17.28 ± 0.33^a^
**High producers (Morning milking)**	13.37 ± 0.17^a^	11.27 ± 0.23^a^	3.81 ± 0.06^a^	67.80 ± 2.10 c	17.16 ± 0.36^ac^
**Moderate producers (Afternoon milking)**	13.43 ± 0.15^a^	9.91 ± 0.21 b	3.88 ± 0.06^a^	87.90 ± 2.55^b^	16.98 ± 0.33^ac^
**High producers (Afternoon milking)**	12.79 ± 0.15^b^	10.27 ± 0.19^b^	2.64 ± 0.00 b	72.30 ± 2.28^a^	16.77 ± 0.30^bc^
**Morning milking yield**	13.43 ±0.11^a^	11.24 ± 0.16^a^	3.90 ± 0.05^ac^	72.60 ± 1.53^ac^	17.04 ± 0.24^ab^
**Afternoon milking yield**	13.07 ± 0.11^ab^	10.12 ± 0.14^b^	3.74 ± 0.04^a^	72.30 ± 1.92^ac^	17.01 ± 0.21^ab^
**Daily milking yield**	13.25 ± 0.08^a^	10.67 ± 0.10^a^	3.84 ± 0.04^a^	76.20 ± 1.17^a^	17.07 ± 0.15^ab^

abc Different letters in each column were significant different (*p *< 0.05).

The diurnal comparison of mean macro-minerals (ANOVA) in the moderate and high producer cows in the morning, afternoon and daily milking yield revealed significant differences (*p* < 0.05) among macro-minerals (Table 2). The same differences (*p* < 0.05) were also observed in individual investigation among macro-minerals in all groups and milking times. 

**Table 2:** Mean diurnal comparison of milk macro-minerals in the moderate and high producer cows in the morning, afternoon and dairy milk yields.

**Parameters**	**Calcium**		**Phosphorus**		**Magnesium**		**Sodium**		**Potassium**	
	**df**	**F**	**df**	**F**	**df**	**F**	**df**	**F**	**df**	**F**
**Moderate producers (Morning milking)**	6(234)	17.6**	6(236)	16.8**	6(239)	10.6**	6(233)	3.6**	6(239)	7.2**
**Moderate producers (Afternoon milking)**	6(234)	17.9**	6(236)	13.8**	6(239)	17.6**	6(234)	70.0**	6(234)	11.1**
**High producers (Morning milking)**	6(249)	30.3**	6(49)	36.9**	6(239)	31.2**	6(239)	10.6**	6(216)	11.0**
**High producers (Afternoon milking)**	6(264)	14.1**	6(262)	25.5**	6(254)	11.3**	6(252)	5.1**	6(264)	9.5**
**Morning milking yield**	6(484)	31.1**	6(485)	52.7**	6(502)	39.1**	6(489)	6.1**	6(491)	22.4**
**Afternoon milking yield**	6(502)	30.0**	6(502)	64.8**	6(494)	25.5**	6(502)	47.4**	6(507)	14.2**
**Daily milking yield**	6(988)	55.7**	6(988)	104.3**	6(1003)	42.7**	6(985)	13.9**	6(990)	16.8**
**Overall**	13(988)	55.7**	13(988)	60.9**	13(1003)	29.8**	13(985)	7.8**	13(990)	32.3**

** indicates significant different (*p* < 0.01); df = degree of freedom, F = F value.

There were significant positive correlations (*p* < 0.05) between the macro-minerals in both producers groups and overall except for milk Na^+^ and K^+^, in which the relationship was negative. The strongest correlation (r= 0.37, *p* < 0.05) was observed between Ca^++^ and IP, while the weakest was between IP and Na^+^ (r= 0.10, *p* < 0.05) (Table 3).

**Table 3:** Correlations among milk macro-minerals concentrations in the morning, afternoon and daily milking in cows.

**Parameters **	**Phosphorus**		**Magnesium**		**Sodium**		**Potassium**	
	**df**	**r**	**df**	**r**	**df**	**r**	**df**	**r**
	**Afternoon milking**							
**Ca** ^++^	502	0.68**	503	0.53**	476	0.19**	480	0.21**
**IP**			503	0.45**	475	0.14**	480	0.22**
**Mg** ^++^					484	0.20**	489	0.11*
**K** ^+^							501	0.17**
	**Morning milking**							
**Ca** ^++^	452	0.45**	459	0.30**	447	0.05	449	0.24**
**IP**			462	0.44**	452	0.12*	454	0.23**
**Mg** ^++^					482	0.25**	481	0.43*
**K** ^+^							489	-0.19**
	**Daily milking**							
**Ca** ^++^	928	0.37**	942	0.24**	925	0.11**	929	0.26**
**IP**			942	0.30**	924	0.10**	928	0.26**
**Mg** ^++^					980	0.21**	983	0.32*
**K** ^+^							990	-0.33**

** indicates significant different (*p* < 0.01),

* indicates significant different (*p* < 0.05); df = degree of freedom, r = correlation coefficient.

## Discussion

In order to increase the benefits of cattle production, special consideration has been given to the macro-mineral administration in animal food during the growth phase, pregnancy and lactation periods. Determination of macro-minerals and their relationships in blood, urine^9^ and CSF^3^ has been successfully reported by some authors, while in milk it needs further investigation. The level of Ca^++^ in milk was recorded over 15 times of that blood,^1^ and thus it would be the best source for young newborn calves' growth. Meanwhile, milk Ca^++^ originated from blood and the increase in the excretion of Ca^++^ into milk resulted in hypocalcaemia or milk fever.^3^ According to the literature, the milk Ca^++^ was not stable,^5^ and monthly and seasonal variations were also reported;^10^ however, it need further investigation during the milking times per day. Otherwise, the findings of this study will support the theory that the physiology of macro-minerals in milk is similar to blood, urine and CSF. Finally, these results could lead to the appropriate use of mineral supplementation for growth, production and reproduction performance, and prevention of metabolic diseases as well. 

In this study the mean daily milk Ca^++^ and IP were 6 and 8 fold that of blood, respectively, which was less than (22 and 17 folds of blood) that recorded by others.^1^^,^^10^ This shows that the concentrations of milk minerals were not stable and varied based on nutrition,^11^ breed,^4^ lactation period, milk protein and urea and milk yield.^12^ It was shown that concentrate feeding, including 1% minerals accompanied by alfalfa resulted in increased milk Ca^++^ and IP, while high protein and urea in food decreased the mentioned minerals in the milk. Milk Ca^++^ was reported to be higher in high producer cows than in moderate ones in that it was not in agreement with this study.^8^ The variation in human and cows' milk Ca^++^ was arranged by the level of milk citrate and casein, thus the presence of these compounds is necessary for Ca^++^ stabilization in the milk.

The amount of milk Ca^++^ absorption from gut depends directly on milk Mg^++^. Although the amount of Ca^++^ is 10 times that of Mg^++^ in milk, the absorption rate for both minerals from the gut is the same. This means that a huge amounts of milk Ca^++^ is not absorbed due to low milk Mg^++^, therefore, adding Mg^++^ in milk resulted in a high absorption of milk Ca^++^ from gut.^1^ Milk Ca^++^ and IP play a role in the formation of bones in calves, and lack of these minerals causes malformation of the skeletal system and rickets.^1^ A high level of Ca^++^ and IP in the milk of healthy cows is an appropriate point in milk production and it can appear at the beginning and the end of lactation periods, while in mastitis they will sharply decline to the lowest level.^5^ Feeding of sodium zeolite will help the quality of milk by increasing the Ca^++^ and IP in milk.

Mean milk Na^+^ and K^+^ concentrations in this study were 76.20 and 17.10 mmol L^-1^, in which Na^+^ was higher and K^+^ lower than that reported by other studies.^10^^,^^13^ The variations for these minerals between the morning and afternoon milking were not significant, but the milk Na^+^ in moderate producer cows was greater and milk K^+^ was lower than in high producer cows. Therefore, milk Na^+^ and K^+^ were not stable in the milk as mentioned for milk Ca^++^ and IP, too. The role of milk Na^+^ and K^+^ in providing the necessary minerals for calf growth is unclear, thus there is no reason for a high or low level of these minerals in the milk. In this study the concentration of Na^+^ in milk was higher than K^+^, and as only 1% of milk dry matter is its minerals, the low milk K^+^ results in an increase in milk Ca^++^ or Na^+^ in that they are considered as an appropriate point in the quality of milk. There is no reports to show the side effects of low milk K^+^ or low absorption from the gut, but milk K^+^ and salts increase following mastitis,^14^ while milk Na^+^ decreases in cows estrus.

Mean milk Mg^++^ in this study was 3.84 mmol L^-1^and it was lower (9.00 mmol L^-1^) than that of other reports.^1^^,^^13^ Magnesium in milk is always constant and is about 0.12 g L^-1^, even in hypomagnesaemia and diet with low Mg^++^. Low milk Mg^++^ was reported following high milk yield,^8^ mastitis,^14^ and bovine viral leukemia,^15^ while milk with high Mg^++^ means the high quality of milk, increased milk Ca^++^ absorption in gut, and the prevention of calf milk tetany.^2^ The Mg^++^ content of milk in high milk producer cows is reported around 3.00 to 4.00 g per day in that it depends on the level of Mg^++^ in food, milk yields and disease outbreaks. High milk Mg^++^ shows that the animals' diet and concentrate feeding contain enough Mg^++^ and vice versa, thus in the latter situation, correction of cow's nutrition must be considered, as it appears necessary for this study, too. Milk Mg^++^ in the morning milking and moderate producer cows was higher than that the afternoon milking and high producer cows. The reason could be related to the resting time between the two milking, and the high milk dry matter in moderate producer cows in comparison with high producers. The same result was reported by Gabris and Bajan in low and high producer cows.^8^

Mean milk macro-minerals in the morning, afternoon and daily milking (Table 2) were significantly different and it was in agreement with similar studies based on the monthly and seasonal milk variations.^8^ The reason for the differences between milking times could be related to the variations in the amounts of feed consumption between cows and milk volume as well. These variations involved all macro-minerals including Ca^++^, IP, Mg^++^, Na^++^ and K^+^,^5^ but the importance of Ca^++^ and Mg^++^ is more apparent than other minerals. 

The presence of significant positive correlations between milk macro-minerals in the morning, afternoon and daily milk yield indicates their appropriate balances in milk production among healthy animals as mentioned for blood, the musculoskeletal system and CSF minerals by authors. The strong relationships between Ca^++^/IP (r = 0.88), Ca^++^/Mg^++^ (r = 0.88) and Na^+^/K^+^ (r = 0.88) presented in this study indicate that the proportion of these minerals in the diet could be vital for animal milk yield. The research related to this subject limited only to the surveys of Gabris and Bajan in that the correlations between Ca^++^, Na^+^ and K^+^ were reported only in high producer cows.^8^

It is concluded that the milk macro-mineral concentrations varied from different milk producer cows to various milking times. Milk Na^+^ was the highest while Mg^++^ was the lowest in cow's milk. Milk Ca^++^ and IP showed the highest positive correlation, while milk Na^++^ and K^+^ showed the negative correlation. Thus, through balancing the macro-minerals in animals' diets, it may be possible to achieve the highest milk quality and quantity yield.
